# Clinical Clerks in Infirmaries

**Published:** 1913-05-24

**Authors:** 


### CLINICAL CLERKS IN INFIRMARIES.

By H. B. M.

The appointment of clinical clerks is a common feature of Scottish institutional life. In any of the large buildings which are at times liable to be overworked the assistance of senior medical students is solicited as clinical clerks. This is especially the custom in most of the Scottish mental hospitals, more particularly those near the university centres. Clinical clerks are to be found in the Poor-Law institutions as well as in those for paying patients (the Royal Asylums). In this way close association is maintained between these institutions and the universities and the medical training schools. The appointments are usually made to those students who have distinguished themselves during their career at the university, or in other ways given evidence of close application to their work. Clinical experience in mental cases, a knowledge of institutional life, and contact with medical graduates are the benefits accruing to these students; while in conversation and by their work they promulgate the latest idea of their up-to-date tutors. The cost of their upkeep is very small. There is no pay attached to the position, only emoluments of board and lodging. The additional commissariat entails comparatively little expenditure.

In this way many a poor but hard-working or brilliant undergraduate has been enabled to pave his way to future success. One of the greatest drawbacks of the London Poor-Law infirmaries is their isolation from the stimulating work of the general hospitals; there is no connecting link between them. The general hospital sends its chronic and perhaps hopeless cases to the infirmaries in order to make room for more acute or interesting patients; and the idea obtains that all the patients in the Poor-Law infirmaries are the chronic or the moribund. The appointment of resident clinical clerks in the infirmaries would tend to draw these two classes of medical institutions more closely and enable the one to appreciate more fully the value of the other. The work of such clerks would be decided by the medical superintendent. It would include the reporting of the specially interesting cases, the testing of excreta, the taking of blood counts, etc. Slowly but surely the good, often excellent, work done in the infirmaries would filter to the general hospitals; the student—the future medical practitioner—will carry away a correct impression of the value of the institution; the resident medical officers will gain from the contact of the enthusiastic clinical, kept informed of the latest developments in medicine, and stimulated by the reports of the latest work in the general hospitals. The idea has been found to produce excellent results in Scotland, and is worthy of a trial in the Metropolis. At least two clinical clerks should be appointed to each infirmary, and facilities given for attendance at their hospital classes. This is one of the ways in which the general hospital's contempt for infirmaries might be modified, and any prejudice against the institution be overcome.

